# Enhancing oxygen and hydrogen evolution activities of perovskite oxide LaCoO_3_*via* effective doping of platinum†

**DOI:** 10.1039/c9ra05491j

**Published:** 2019-11-04

**Authors:** Caiyun Wang, Lirong Zeng, Wei Guo, Cairong Gong, Jing Yang

**Affiliations:** School of Materials Science and Engineering, Tianjin University Tianjin 300072 P. R. China gcr@tju.edu.cn yang_jing@tju.edu.cn

## Abstract

In this study, a series of perovskite oxides LaCo_1−*x*_Pt_*x*_O_3−*δ*_ (*x* = 0, 0.02, 0.04, 0.06, and 0.08) were prepared by the citric acid–ethylenediaminetetraacetic acid (CA–EDTA) complexing sol–gel method and characterized by X-ray diffraction (XRD), scanning electron microscopy (SEM), transmission electron microscopy (TEM) and X-ray photoelectron spectroscopy (XPS). Then, the samples were investigated as OER and HER bifunctional electrocatalysts in alkaline media. Compared with other catalysts, LaCo_0.94_Pt_0.06_O_3−*δ*_ had good stability and presented more activity at a lower overpotential of 454 mV (at 10 mA cm^−2^), a lower Tafel slope value of 86 mV dec^−1^ and a higher mass activity of 44.4 A g^−1^ for OER; it displayed a lower overpotential of 294 mV (at −10 mA cm^−2^), a lower Tafel slope value of 148 mV dec^−1^ and a higher mass activity of −34.5 A g^−1^ for HER. The improved performance might depend on a larger ECSA, faster charge transfer rate and higher ratio of the highly oxidative oxygen species (O_2_^2−^/O^−^). Furthermore, the e_g_ orbital filling of Co approaching 1.2 in the B site might play a leading role.

## Abstract 2

Among the perovskite LaCo_1−*x*_Pt_*x*_O_3−*δ*_ catalysts, LaCo_0.94_Pt_0.06_O_3−*δ*_ proved best for catalyzing OER/HER, with *η* = 454/294 mV, which might be attributed to LCP6 having the e_g_ orbital filling of Co closest to 1.2.

## Graphical abstract

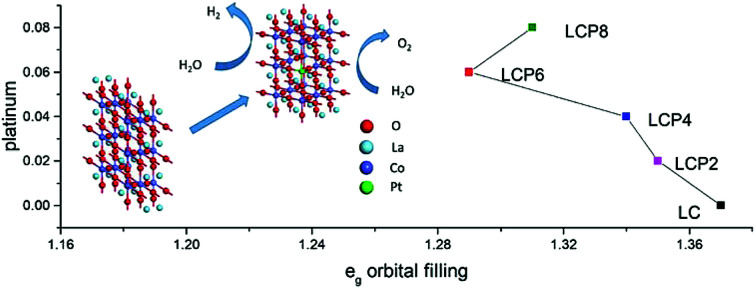

## 1. Introduction

With the rapid depletion of fossil fuels and the resulting environmental problems, researchers are working hard to search for sustainable alternative energy sources and energy storage and changeover methods.^1^ As a result, electrochemical water splitting, regarded as promising due to its simplicity and environmental friendliness for energy storage and changeover, has attracted great interest.^2–4^ However, because of the sluggish kinetics of the two vital half-reactions, oxygen evolution reaction (OER) on the anode and hydrogen evolution reaction (HER) on the cathode, for electrochemical water splitting in alkaline environment, electrocatalytic materials are needed to hasten the two half-reactions.^5^ Although at the present stage, precious metal oxides IrO_2_/RuO_2_ and precious metal Pt-based materials have been recognized as state-of-the-art electrocatalysts for OER and HER, respectively, limited resources and high costs have restricted their wide-ranging application.^6^ Therefore, researchers have developed non-noble metal nitrides,^7,8^ phosphides,^9^ carbides,^10^ borides,^11^ and oxides to replace noble metal electrocatalysts for OER or HER and have achieved good results. Among them, non-noble metal perovskite oxides, with plentiful reserves, low cost, excellent redox performance and stable structure, have gradually become the topic of interest for researchers all over the world.^12^

Compared with other types of oxides, perovskite oxides have flexible structure and composition, with a general formula of ABO_3_, where the larger cation A is commonly a lanthanide, rare-earth metal or alkaline earth metal which has 12-fold coordination with O^2−^ anions and the smaller cation B is a transition metal in corner-sharing octahedra with O^2−^ anions. The composition and structure lead to easily doping elements or making deficiencies in A or B sites, which greatly affect the valence and structure of the transition metals, further improving the OER and HER performances of the materials.^13–17^ Shao and Haile confirmed that the doped perovskite Ba_0.5_Sr_0.5_Co_0.8_Fe_0.2_O_3−*δ*_ (BSCF) showed excellent OER activity in alkaline conditions, even exceeding that of IrO_2_.^13^ Afterward, BSCF and Pr_0.5_BSCF were also proved to be good electrocatalysts for HER.^14^ In addition, oxygen vacancies caused by the changing valence of the transition metals in perovskite catalysts such as BaTiO_3−*δ*_,^18^ SrCoO_3−*δ*_,^19^ and La_1−*x*_Sr_*x*_CoO_3−*δ*_ (ref. 20) could act as the active sites to influence the electrocatalytic performance. Suntivich *et al.* systematically studied a dozen perovskite oxides and sketched a volcano map of the relationship between the intrinsic OER activity of perovskite oxides and the e_g_ orbital filling: when the number of e_g_ orbital is about 1.2, the catalyst will display a good OER activity.^21^ Many articles have confirmed the correctness of the e_g_ orbital filling descriptor.^22–24^ In addition, recent studies have demonstrated that the e_g_ orbital filling descriptor is also valid for HER.^25^ Grimaud *et al.* highlighted that the distance of the O p-band center relative to the Fermi level could be used as another descriptor to characterise the OER activity and stability.^26^ Researchers have investigated the composite materials of precious-metal Pt and perovskite oxides, such as Pt/CaMnO_3_,^27^ Pt/C–Ba_0.5_Sr_0.5_Co_0.8_Fe_0.2_O_3−*δ*_ and Pt/C-PrBaCo_2_O_5+d_.^28^ They proposed that some kind of synergistic effect between precious-metal Pt and perovskite oxides could improve the electrocatalytic performance of perovskite oxides.^27–29^ Although the above research has continued, the overpotential of perovskite oxide electrocatalysts is still unsatisfactory. Furthermore, there have been few perovskite oxides studied as OER and HER bifunctional electrocatalysts.^15–17,30–32^

In the present paper, we report our findings on enhancing OER and HER activities by doping minor precious metal Pt into perovskite oxide LaCoO_3_ in an alkaline condition. LaCo_1−*x*_Pt_*x*_O_3−*δ*_ (*x* = 0, 0.02, 0.04, 0.06, 0.08) perovskite (denoted as LC, LCP2, LCP4, LCP6, and LCP8, respectively) were prepared *via* the citric acid–ethylenediaminetetraacetic acid (CA–EDTA) complexing sol–gel method. Among these doped perovskite oxides, LaCo_0.94_Pt_0.06_O_3−*δ*_ (LCP6) shows the highest OER and HER activities, demonstrating that LaCo_0.94_Pt_0.06_O_3−*δ*_ (LCP6) could be a promising candidate as an OER and HER bifunctional electrocatalyst in an alkaline condition.

## 2. Experimental section

### 2.1. Chemicals

La(NO_3_)_3_·6H_2_O (99.99%, AR grade), Co(NO_3_)_2_·6H_2_O (99.99%, AR grade), KOH (99.98%, AR grade), citric acid (CA, 99.99%, AR grade), ethylenediaminetetraacetic acid (EDTA, 99.99%, AR grade), and ammonia hydroxide (25%, AR grade) were purchased from Kermel. Pt(NO_3_)_2_ (Pt, 18.02%) was bought from Aladdin. Nafion solution (5 wt%, v : v : v = 4 : 1 : 0.04) was bought from Sigma-Aldrich. Carbon black (CB, VXC-72) was purchased from Cabot, USA. All chemicals were used directly without further purification.

### 2.2. Synthesis of LCP

A series of perovskite oxides LaCo_1−*x*_Pt_*x*_O_3−*δ*_ with *x* = 0, 0.02, 0.04, 0.06 and 0.08 was synthesized *via* the CA–EDTA complexing sol–gel method. Stoichiometric La(NO_3_)_3_·6H_2_O, Co(NO_3_)_2_·6H_2_O and Pt(NO_3_)_2_ were dissolved in deionized water to form a clarified purple solution, with CA and EDTA added to the above solution in the molar ratio of total metal ions : CA : EDTA = 1 : 2 : 1. Then 25% ammonia hydroxide was dropped into the stirring solution until pH = 6.0–7.0. The mixture was magnetically stirred in water at 80 °C to form a transparent purple wet gel, followed by drying at 120 °C for 24 h. The obtained xerogel was calcined at 400 °C for 3 h to remove organic impurities and then at 850 °C for 5 h to form the final perovskite structure.

### 2.3. Catalyst characterizations

Powder X-ray diffraction (XRD) was recorded on a D8 Advance diffractometer employing Cu Kα radiation (*λ* = 1.5406 Å) in the range of 20–80° with a scan rate of 6° min^−1^. Scanning electron microscopy (SEM) was carried out on a JEOL JSM-7800F thermal field emission scanning electron microscope with an accelerating voltage of 15.0 kV. Transmission electron microscopy (TEM) was performed on a JEOL JEM-2100F field emission transmission electron microscope with a 200.0 kV accelerating voltage. X-ray photoelectron spectra (XPS) were examined by a Kratos-Axis Ultra DLD photoelectron spectrometer equipped with an Al Kα (*hν* = 1486.6 eV) X-ray source.

### 2.4. Electrode preparation

5 mg catalyst powder, 1 mg carbon black after nitric acid treatment, 450 μL deionized water, 300 μL isopropanol and 50 μL Nafion solution were mixed to form ink, followed by ultrasonication for 2 h. Then, 7.2 μL of the catalyst ink was dropped onto an active carbon fiber paper (0.5 cm × 0.3 cm) pre-treated in nitric acid to clean the surface. The active carbon fiber paper coated with ink was air dried in the surrounding environment for 2 h. The catalyst mass loading on the carbon fiber paper was nearly 0.3 mg cm^−2^. Furthermore, the catalyst mass loading on the carbon fiber paper in the overall water splitting test was nearly 0.6 mg cm^−2^.

### 2.5. Electrochemical measurements

Electrochemical measurements were performed in a conventional three-electrode electrochemical cell using a Hg/HgCl_2_ electrode in saturated KCl solution as the reference electrode and a Pt wire for OER and a graphite rod for HER as the counter electrodes. The supporting electrolyte was 0.1 M aqueous KOH solution. The polarization curves were recorded using linear sweep voltammetry (LSV) under potential windows from 0 to 1.0 V and −1.8 to −0.9 V (*vs.* Hg/HgCl_2_) for OER and HER, respectively, with a scan rate of 5 mV s^−1^. The overall water splitting was tested by a two-electrode electrochemical cell in 0.1 M aqueous KOH solution at a scan rate of 5 mV cm^−2^ with the mass loading of catalyst at 0.6 mg cm^−2^. The double layer capacitance (Cdl) was acquired *via* the cyclic voltammetry (CV) method with different scan rates of 5, 20, 40, 60 and 80 mV s^−1^ and a potential window from 0.3 to 0.4 V (*vs.* Hg/HgCl_2_), in which no faradaic processes happened. Slopes of the curves between half of the anodic and cathodic current difference centered at 0.35 V (*vs.* Hg/HgCl_2_) and scan rates represented the double layer capacitance.^5^ Electrochemical impedance spectroscopy (EIS) was carried out at 0.7 V and −1.5 V (*vs.* Hg/HgCl_2_) for OER and HER, respectively, within a frequency range of 0.1 to 105 Hz. The overall water splitting test was carried out in a two-electrode system.

## 3. Results and discussion

### 3.1. Characterization of the catalysts

**Fig. 1:**
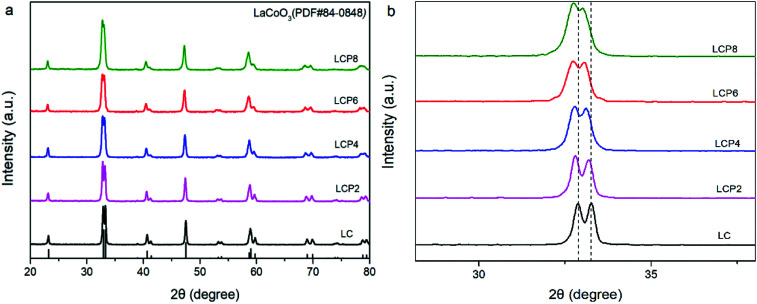
(a) XRD patterns of LaCo_1−*x*_Pt_*x*_O_3−*δ*_ (*x* = 0, 0.02, 0.04, 0.06, 0.08) powders. (b) Local amplification of the XRD patterns at 30–35°.

Fig. 1a shows the X-ray diffraction (XRD) patterns of the as-prepared LC, LCP2, LCP4, LCP6 and LCP8 powders. It can be observed that all diffraction peaks are consistent with the standard pattern of LC (JCPDS no. 84-0848) without any impurity peaks, implying high quality synthesized products. However, as shown in the local amplification in Fig. 1b, the two main diffraction peaks (∼32.9° and ∼33.3°) obviously shifted to the low-angle region with the increase of platinum doping amount. That reflects the expansion of the lattice parameters, which may be attributed to the larger radius of platinum ion than cobalt ion. Additionally, no peaks of platinum or platinum oxides were found in the XRD patterns, which may be due to platinum entering the perovskite lattice entirely or minor platinum doping being difficult to detect. Furthermore, the two main diffraction peaks tended to gradually merge into one peak with the increase of platinum doping amount (Fig. 1b), implying distortion of the tripartite phase to cubic.

**Fig. 2:**
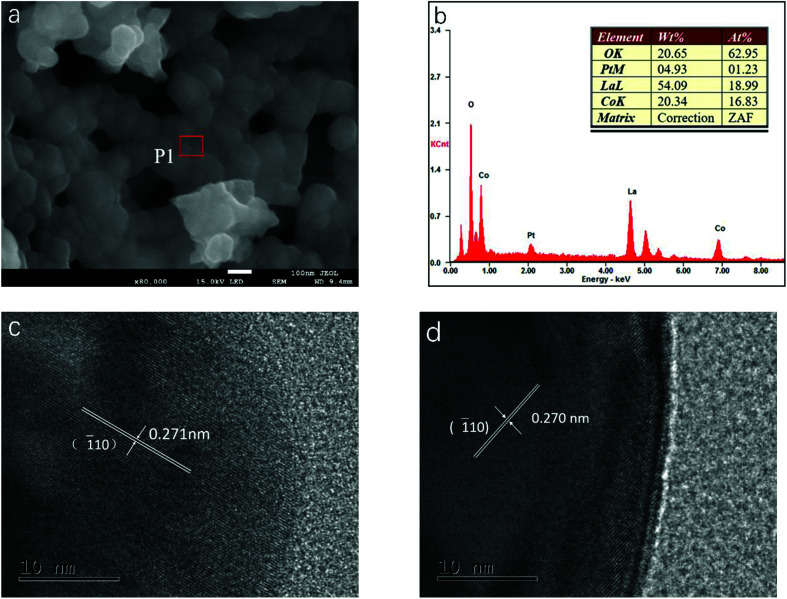
(a) SEM image of LaCo_0.94_Pt_0.06_O_3−*δ*_ catalyst. (b) EDX spectrum measured at position P1 in (a). (c and d) HRTEM images of LaCo_0.94_Pt_0.06_O_3−*δ*_ and LaCoO_3_ catalysts, respectively.

The particulate morphologies of the samples were investigated by SEM (Fig. 2a and S1, ESI†). All of them revealed particles with a grain size of about 100 nm. Fig. 2b showed the EDX spectrum obtained for LCP6, which displayed the surface elements of the product. The molar ratio of surface elements La, Co and Pt calculated by mass fraction and atomic fraction is about 1 : 0.89 : 0.06, which is close to the stoichiometric ratio of LCP6. As displayed in Fig. 2c and d, HRTEM images exhibited lattice fringe spacings of *d* = 0.271 nm and 0.270 nm for LCP6 and LC, respectively, corresponding to the (1̄10) crystal plane of the perovskite structure (JCPDS no. 84-0848). Furthermore, no lattice fringes of platinum or platinum oxides were found, which corresponded to the XRD patterns. The above results indicated the successful synthesis of doped perovskite.

**Fig. 3:**
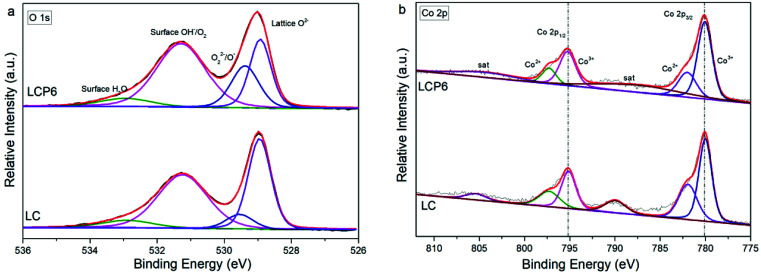
XPS spectra of (a) O 1s and (b) Co 2p on the surface of LaCo_0.94_Pt_0.06_O_3−*δ*_ and LaCoO_3_ catalysts.

The species on sample surfaces were examined through high-resolution X-ray photoelectron spectroscopy (XPS). As shown in Fig. 3a and S3a (ESI),† the XPS spectra of O 1s were deconvoluted into four characteristic peaks at ∼528.9 eV (lattice oxygen O^2−^), ∼529.6 eV (highly oxidative oxygen species for O_2_^2−^/O^−^), ∼531.3 eV (hydroxyl groups or surface adsorbed oxygen for –OH or O_2_) and 532.9 eV (adsorbed molecular water for H_2_O).^5,14^ The O 1s XPS peak deconvolution results of all prepared catalysts are shown in Table 1. The XPS spectra of Co 2p are shown in Fig. 3b and S3b (ESI).† After deconvolution, the peaks of Co 2p_3/2_ and Co 2p_1/2_ at ∼780.1 and ∼795.2 eV belong to Co^3+^, whereas the peaks of Co 2p_3/2_ and Co 2p_1/2_ at ∼782.0 and ∼797.3 eV belong to Co^2+^.^33^ The ratios of Co^3+^ to Co^2+^ based on peak area intensity are listed in Table 2. The XPS spectra of Pt 4f and the corresponding deconvolutions for 4f_7/2_ and 4f_5/2_ are shown in Fig. S3c in the ESI.† Both Pt 4f_7/2_ and 4f_5/2_ could be deconvoluted to two peaks assigned to Pt^4+^ and Pt^2+^.^34,35^

**Table 1:** O 1s XPS peak deconvolution results

Catalysts	Lattice O^2−^	O_2_^2−^/O^−^	Surface –OH/O_2_	Surface H_2_O
LC	37.53%	7.11%	48.42%	6.94%
LCP2	35.39%	8.98%	46.29%	9.34%
LCP4	31.15%	13.15%	50.91%	4.79%
LCP6	24.05%	18.76%	50.45%	6.74%
LCP8	31.93%	14.10%	46.78%	7.19%

**Table 2:** Co 2p and Pt 4f XPS peak deconvolution results

Catalysts	Co^3+^/Co^2+^	Pt^4+^/Pt^2+^	Average valence of Co	Average valence of Pt	Oxygen vacancies	e_g_ orbital filling
LC	1.69	0	+2.63	0	0.185	1.37
LCP2	1.88	1.05	+2.65	+3.02	0.171	1.35
LCP4	1.98	0.38	+2.66	+2.55	0.172	1.34
LCP6	2.50	0.22	+2.71	+2.37	0.155	1.29
LCP8	2.21	0.47	+2.69	+2.64	0.157	1.31

### 3.2. Electrocatalytic activities of the catalysts for the OER and HER

**Fig. 4:**
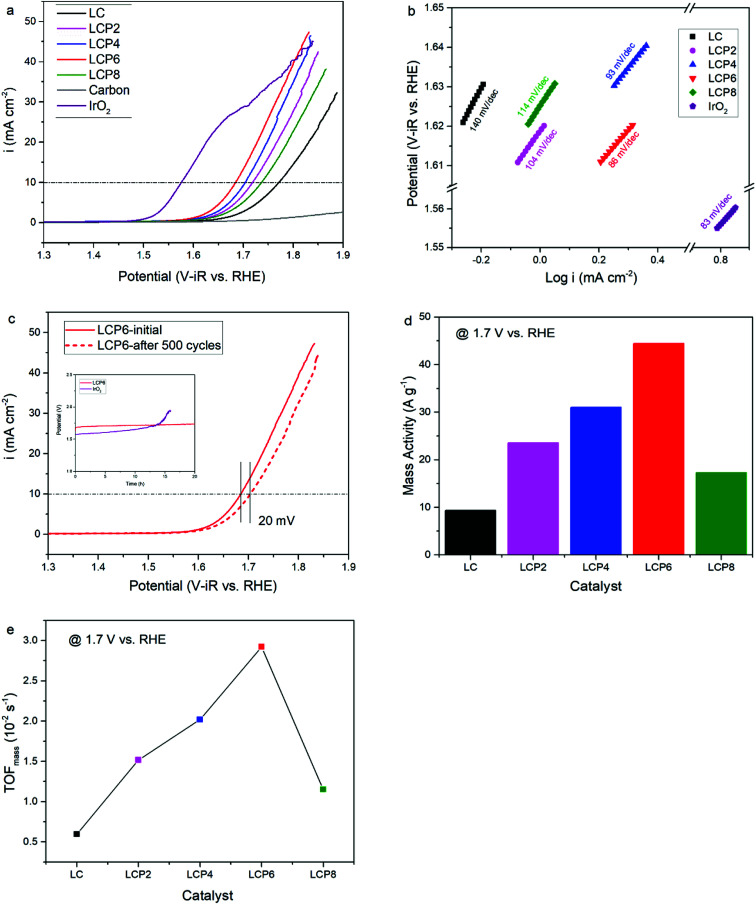
(a) The LSV OER curves and (b) corresponding Tafel plots of LaCo_1−*x*_Pt_*x*_O_3−*δ*_ (*x* = 0, 0.02, 0.04, 0.06, 0.08) and IrO_2_ powders in 0.1 M KOH solution. Scan rate, 5 mV s^−1^. (c) LSV OER curves of LaCo_0.94_Pt_0.06_O_3−*δ*_ catalyst initially and after 500 cycles in the chronopotentiometry tests of LaCo_0.94_Pt_0.06_O_3−*δ*_ and IrO_2_ for 20 h in 0.1 M KOH solution. (d) Mass activity and (e) TOF_mass_ data of LaCo_1−*x*_Pt_*x*_O_3−*δ*_ (*x* = 0, 0.02, 0.04, 0.06, 0.08) powders at 1.7 V *vs.* RHE.

As shown in Fig. 4a, the OER activity of the catalysts was tested first by linear sweep voltammetry (LSV) at a scan rate of 5 mV s^−1^ in 0.1 M KOH aqueous solutions at room temperature. Compared with other samples, the OER polarization curve of LCP6 exhibited the lowest onset potential of ≈1.59 V *vs.* RHE, implying a better start of LCP6 for catalyzing OER. Because of the connection to solar-to-fuels conversion, the overpotential (*η*) at the current density of 10 mA cm^−2^ is an important parameter for solar cells.^36^ Obviously, the LCP6 showed the lowest overpotential of 454 mV at the current density of 10 mA cm^−2^, followed by LCP4 (474 mV), LCP2 (488 mV), LCP8 (507 mV) and LC (541 mV), suggesting the best OER activity of LCP6. In addition, the Tafel slope (Fig. 4b) for LCP6 (86 mV dec^−1^) was the smallest among all the samples, presenting the enhanced OER activity of LCP6 over the others. In addition to activity, stability is equally important for electrocatalysts. LCP6, which has the lowest overpotential, was tested by cyclic voltammetry for 500 cycles at a scan rate of 50 mV s^−1^ and by chronopotentiometry at the current density of 10 mV cm^−2^ continuously for 20 h to evaluate stability. After cycling, the linear sweep volt–ampere curve only showed a minor voltage increase of about 20 mV at the current density of 10 mA cm^−2^ (Fig. 4c). The chronopotentiometry test (illustration in Fig. 4c) showed that the overpotential of LCP6 hardly changed during the test, which illustrated the great stability of LCP6 for catalyzing OER. Although the overpotential of LCP6 is larger than that of IrO_2_ (346 mV in Fig. 4a), chronopotentiometry showed that the stability of LCP6 is much higher than that of IrO_2_. Fig. 4d compared the mass activities of all the materials at 1.7 V (*vs.* RHE). LCP6 had the highest value of 44.4 A g^−1^, 4-times higher than that of LC (9.3 A g^−1^), demonstrating that the doped perovskite oxide LCP6 has promising OER activity, while the mass activities of LCP2, LCP4 and LCP8 were 23.6 A g^−1^, 31.0 A g^−1^, and 17.3 A g^−1^, respectively. Next, the turnover frequencies (TOF_mass_) of the catalysts were calculated to understand the improved activity. Based on common sense, only the B-site metals in the octahedral sites of perovskite material were used as the active sites.^37^ Formula (2) in the ESI† was used to calculate the TOF_mass_. As can be seen in Fig. 4e, LCP6 presented the highest TOF_mass_ value of 2.92 × 10^−2^ s^−1^, 4-times higher than that of LC (0.59 × 10^−2^ s^−1^), confirming the excellent OER activity of LCP6 as an electrocatalyst. The TOF_mass_ values of LCP2, LCP4 and LCP8 were 1.52 × 10^−2^ s^−1^, 2.02 × 10^−2^ s^−1^, and 1.15 × 10^−2^ s^−1^ respectively.

**Fig. 5:**
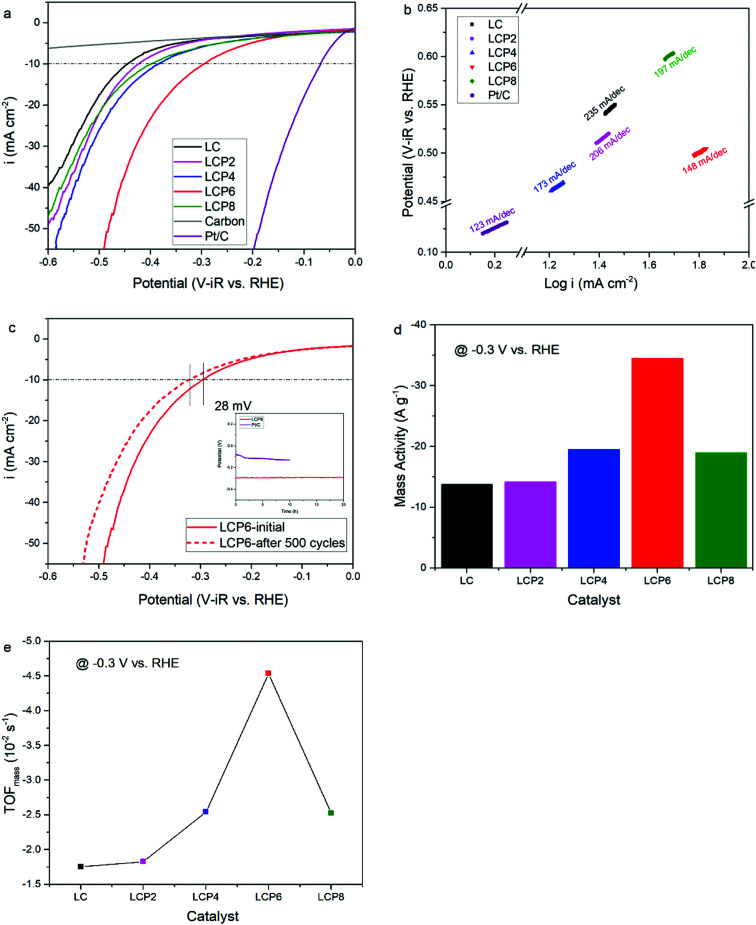
(a) The LSV HER curves and (b) the corresponding Tafel plots of LaCo_1−*x*_Pt_*x*_O_3−*δ*_ (*x* = 0, 0.02, 0.04, 0.06, 0.08) and Pt/C powders in 0.1 M KOH solution. Scan rate, 5 mV s^−1^. (c) LSV HER curves of LaCo_0.94_Pt_0.06_O_3−*δ*_ catalyst initially and after 500 cycles in the chronopotentiometry test of LaCo_0.94_Pt_0.06_O_3−*δ*_ and Pt/C for 20 h in 0.1 M KOH solution. (d) Mass activity and (e) TOF_mass_ data of LaCo_1−*x*_Pt_*x*_O_3−*δ*_ (*x* = 0, 0.02, 0.04, 0.06, 0.08) powders at −0.3 V *vs.* RHE.

The HER activity of the catalysts was examined under the same conditions as the OER activity. Fig. 5a presents the HER polarization curves, from which we could obtain the same conclusion that LCP6, with an onset potential of ≈0.11 V *vs.* RHE, has the best performance to catalyze HER, followed in sequence by LCP4, LCP8, LCP2 and LC. At the current density of −10 mA cm^−2^, the required overpotential for LCP6 was only 294 mV (Fig. 5a), which was the lowest numerical value when compared to LCP4 (384 mV), LCP8 (398 mV), LCP2 (423 mV) and LC (444 mV). Furthermore, the smallest Tafel value of 148 mV dec^−1^ for LCP6 (Fig. 5b) relative to other samples was observed. The stability of LCP6 for HER was also measured by cyclic voltammetry for 500 cycles at a scan rate of 50 mV s^−1^ and a chronopotentiometry test at the current density of 10 mV cm^−2^ for 20 h. As shown in Fig. 5c, the linear sweep volt–ampere curve also showed merely a minor voltage increase of about 28 mV at the current density of −10 mA cm^−2^ after 500 cycles and the almost unchanged overpotential in the chronopotentiometry test demonstrated that LCP6 has an excellent stability. Although the overpotential of LCP6 is larger than that of Pt/C (66 mV, Fig. 5a), chronopotentiometry showed that the stability of LCP6 is much higher than that of Pt/C. The mass activity (−34.5 A g^−1^ at −0.3 V *vs.* RHE, Fig. 5d) and the TOF_mass_ value (−4.54 × 10^−2^ s^−1^, Fig. 5e) of LCP6 were the highest among all the as-prepared perovskite oxides, proving the outstanding HER activity of LCP6.

**Fig. 6:**
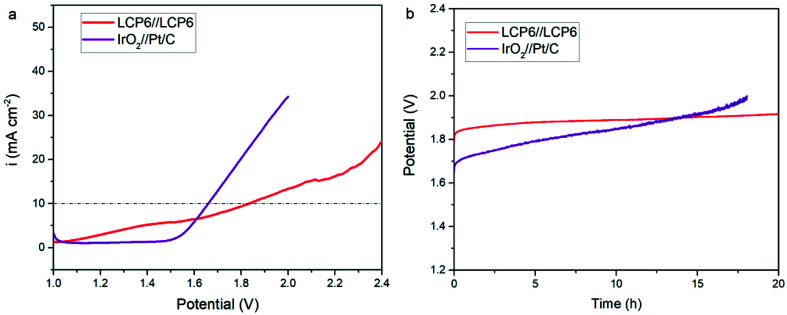
(a) Polarization curves of LCP6//LCP6 and IrO_2_//Pt/C for overall water splitting in 0.1 M KOH solution. Scan rate, 5 mV s^−1^. (b) Chronopotentiometry curves for water electrolysis of LCP6//LCP6 and IrO_2_//Pt/C at current density of 10 mV cm^−2^.

Fig. 6a displays the results of LCP6//LCP6 for the overall water splitting test. LCP6 was used as both the anode and cathode. When the current density was 10 mV cm^−2^, the required potential for overall water splitting was 1.83 V. Chronopotentiometry tests (Fig. 6b) at the current density of 10 mV cm^−2^ for 20 h continuous operation exhibited the excellent stability of LCP6.

**Fig. 7:**
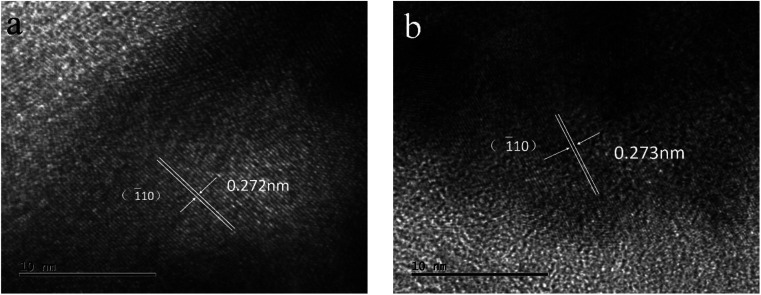
HRTEM images of LaCo_0.94_Pt_0.06_O_3−*δ*_ after cyclic voltammetry for 500 cycles in (a) OER and (b) HER.

After cyclic voltammetry for 500 cycles, the sample of LCP6 was examined by TEM, as shown in Fig. 7 and S4.† HRTEM images exhibited lattice fringe spacings of *d* = 0.272 nm and 0.273 nm in OER and HER, respectively, after the 500 cycles, similar to the lattice fringe spacing of *d* = 0.271 nm before the tests. The overall micro-morphology (Fig. S4†) remained unchanged before and after the tests, which confirmed the structural stability of LCP6.

### 3.3. Factors for the enhanced performance

From the results presented above, LCP6 has proven to be a promising OER and HER bifunctional electrocatalyst. As is well-known, the widely accepted OER mechanism on perovskite oxides in alkaline media is the proposed adsorbate evolution mechanism (AEM).^21,38,39^ Generally, the AEM takes place through four single electron charge transfer steps, involving adsorption and desorption of a sequence of reaction intermediates, O*, OH* and OOH* (where * denotes the surface active site).^5,16^ The overall response begins with the adsorption of OH^−^ and finishes with the desorption of the OH^−^. In contrast, HER proceeds through two steps.^31,40^ The first step is a Volmer reaction (H_2_O + e^−^ → H_ads_ + OH^−^), which is the reduction of water to H atoms and OH^−^ species, then the intermediates are adsorbed onto the surface of the catalysts. The second step is either a Heyrovsky reaction (H_2_O + H_ads_ + e^−^ → H_2_ + OH^−^) or Tafel reaction (H_ads_ + H_ads_ → H_2_), where the former is an electrochemical desorption step and the latter is a chemical desorption one. The Tafel slopes are 120, 40 and 30 mV dec^−1^ when the HER is determined by the Volmer, Heyrovsky and Tafel processes, respectively.^31^ As shown in Fig. 4b, the Tafel slope values of all the investigated samples are close to 120 mV dec^−1^, indicating that the Volmer reaction might be the determining step of HER catalysis.

The XRD patterns showed that the prepared perovskite particles exhibit a crystalline phase transition from tripartite phase to cubic phase with the increase of platinum doping amount. A previous paper proposed that perovskite oxides with a cubic phase may present enhanced electrocatalytic performance.^33^ However, in this work, the crystalline phase may be a secondary factor, because LCP6 showed the best electrocatalytic performance.

**Fig. 8:**
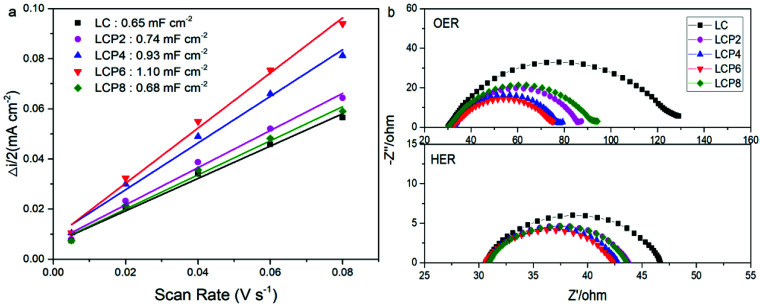
(a) The capacitive currents as a function of scan rate for LaCo_1−*x*_Pt_*x*_O_3−*δ*_ (*x* = 0, 0.02, 0.04, 0.06, 0.08) powders. (b) EIS Nyquist plots of LaCo_1−*x*_Pt_*x*_O_3−*δ*_ (*x* = 0, 0.02, 0.04, 0.06, 0.08) powders recorded at OER potential of 0.7 V (*vs.* Hg/HgCl_2_) and HER potential of −1.5 V (*vs.* Hg/HgCl_2_).

In order to investigate the factors in the enhanced OER and HER activity, we obtained the double layer capacitance (Cdl) of all the catalysts *via* cyclic voltammograms (Fig. S2, ESI†). As shown in Fig. 8a, the Cdl of LCP6 (1.10 mF cm^−2^) was much larger than those of LCP4 (0.93 mF cm^−2^), LCP2 (0.74 mF cm^−2^), LCP8 (0.68 mF cm^−2^) and LC (0.65 mF cm^−2^). Additionally, double layer capacitance is frequently used to reflect the electrochemical active surface area (ECSA) of the catalysts. Thus, the above result suggests that LCP6 has a larger ECSA, indicating a relatively high amount of active sites involved in OER and HER.^41^ The charge transfer resistance during OER and HER was evaluated by the electrochemical impedance spectra (EIS). The Nyquist plots (Fig. 8b) implied that LCP6, with the smallest semicircle diameter compared with other samples, exhibits a lower charge transfer resistance and a faster charge transfer rate, which are beneficial for enhancing electrocatalysis for OER and HER.

To further understand the differences in electrochemical performance, the results of XPS were analysed in depth. From Fig. 3a and Table 1, we can see that LCP6 has a larger ratio of the highly oxidative oxygen species (O_2_^2−^/O^−^) compared with the remaining samples. Previous studies have shown that highly oxidative oxygen species (O_2_^2−^/O^−^) are beneficial to both OER and HER.^21,25,30,42^ From Fig. 3b and Table 2, we get the ratio of Co^3+^ to Co^2+^ of each sample. Generally, the spin state of Co^3+^ is intermediate-spin (IS, e_g_↑3t_2g_↑2t_2g_↓) and Co^2+^ is high-spin (HS, 2e_g_↑3t_2g_↑2t_2g_↓).^24^ Thus, the e_g_ orbital filling of Co was calculated to be 1.29 in LCP6.^22^ Compared with the other samples, the electronic number 1.29 of LCP6 is closest to 1.2. According to the e_g_ orbital filling descriptor theory,^21,25^ LCP6 should have the best electrocatalytic performance for OER and HER. As shown in Fig. S3c,† the Pt 4f_5/2_ peak and 4f_7/2_ peak of LCP6 have shifted to higher binding energy than others, implying the presence of a lower oxidation state of Pt in LCP6. After analysis, the ratio of Pt^4+^/Pt^2+^ and the average valence of Pt in samples are shown in Table 2. The value of LCP6 was the lowest, which corresponded to the peak shift. Table 2 shows that the oxygen vacancies of catalysts decrease due to Pt doping and the number of oxygen vacancies in LCP6 is the lowest. Although the oxygen vacancy is beneficial to the electrocatalytic performance, it is obviously not the main property affecting the performance here. Furthermore, from the mechanisms of OER and HER, it can be seen that the adsorption and desorption of OH^−^ is an important step.^30,33^ Also, the higher oxidation state in the B site is beneficial to the adsorption of OH^−^, which could hasten the initial response of OER and HER. Therefore, LCP6 possessed an enhanced activity.

## 4. Conclusions

In summary, a series of perovskite oxides LaCo_1−*x*_Pt_*x*_O_3−*δ*_ (*x* = 0, 0.02, 0.04, 0.06, 0.08) were prepared by the citric acid–ethylenediaminetetraacetic acid (CA–EDTA) complexing sol–gel method and investigated as OER and HER bifunctional electrocatalysts in alkaline media. The LaCo_0.94_Pt_0.06_O_3−*δ*_ catalyst exhibited the best activity and stability in catalyzing the oxygen and hydrogen evolution reactions with overpotentials of 454 mV and 294 mV (10 mA cm^−2^), respectively, compared with other catalysts. The enhanced performance might be attributed to a larger ECSA, faster charge transfer rate, higher ratio of the highly oxidative oxygen species (O_2_^2−^/O^−^) and the e_g_ orbital filling of Co approaching 1.2 in the B site. In summary, the LaCo_0.94_Pt_0.06_O_3−*δ*_ perovskite is an efficient OER and HER bifunctional electrocatalyst.

## Conflicts of interest

There are no conflicts of interest to declare.

## Supplementary Material

RA-009-C9RA05491J-s001
