# Supplementary material for: Enhancing oxygen and hydrogen evolution activities of perovskite oxide LaCoO3via effective doping of platinum
Source: RSC Adv. 2019 Nov 4;9(61):35646–54. doi: 10.1039/c9ra05491j (PMC9074704; doi:10.1039/c9ra05491j)
Supplement: RA-009-C9RA05491J-s001 [file RA-009-C9RA05491J-s001.pdf]

Electronic Supplementary Material (ESI) for RSC Advances.
This journal is © The Royal Society of Chemistry 2019

Supplementary Materials

Enhancing oxygen and hydrogen evolution activity of perovskite oxide $LaCoO_3$ via effectively doping of platinum

Caiyun Wang, Lirong Zeng, Wei Guo, Cairong Gong*, Jing Yang*

School of Materials Science and Engineering, Tianjin University, Tianjin 300072, P. R. China.
** Corresponding author : E-mail: gcr@tju.edu.cn (C. Gong), yang_jing@tju.edu.cn (J. Yang).*

Turnover frequency (TOF) refers to the number of catalytic reactions per active site per unit time at a given temperature, pressure, reactant ratio and a certain degree of reaction. The TOF value can reflect the intrinsic activity of the catalyst, which can be said to be the best factor to express the intrinsic activity of the catalyst. The formula is

$$TOF(s^{-1}) = \frac{I(A)}{\alpha F\left(As\, mol^{-1}\right) \times\ N(mol)} \quad (1)$$

Among them, I (A) is the value of catalytic current (given directly by electrochemical workstation), F (As mol^{-1}) is Faraday constant, N (mol) is the number of catalytic active sites, and α is the number of transfer electrons corresponding to the semi-reaction of producing or consuming a molecule target product. In OER, α is 4, and in HER, α is 2.

In this paper, we consider that only the B-site metals in the octahedral sites of perovskite material are used as the active sites. Then following Formula (2) was used to calculate the TOF_{mass} at OER potential of 1.7 V (vs. RHE) and HER potential of -0.3 V (vs.RHE).

$$TOF_{mass}\ (\times 10^{-2}\, s^{-1}) = \frac{current\ \left(mA\ cm^{-2}\right) \times\ MW\ of\ LaCo_{1-x}Pt_xO_{3-\delta}\ (g\ mol^{-1})}{\alpha \times F\ \left(As\, mol^{-1}\right) \times\ \ catalyst\ loading\ (mg\ cm^{-2})} \quad (2)$$

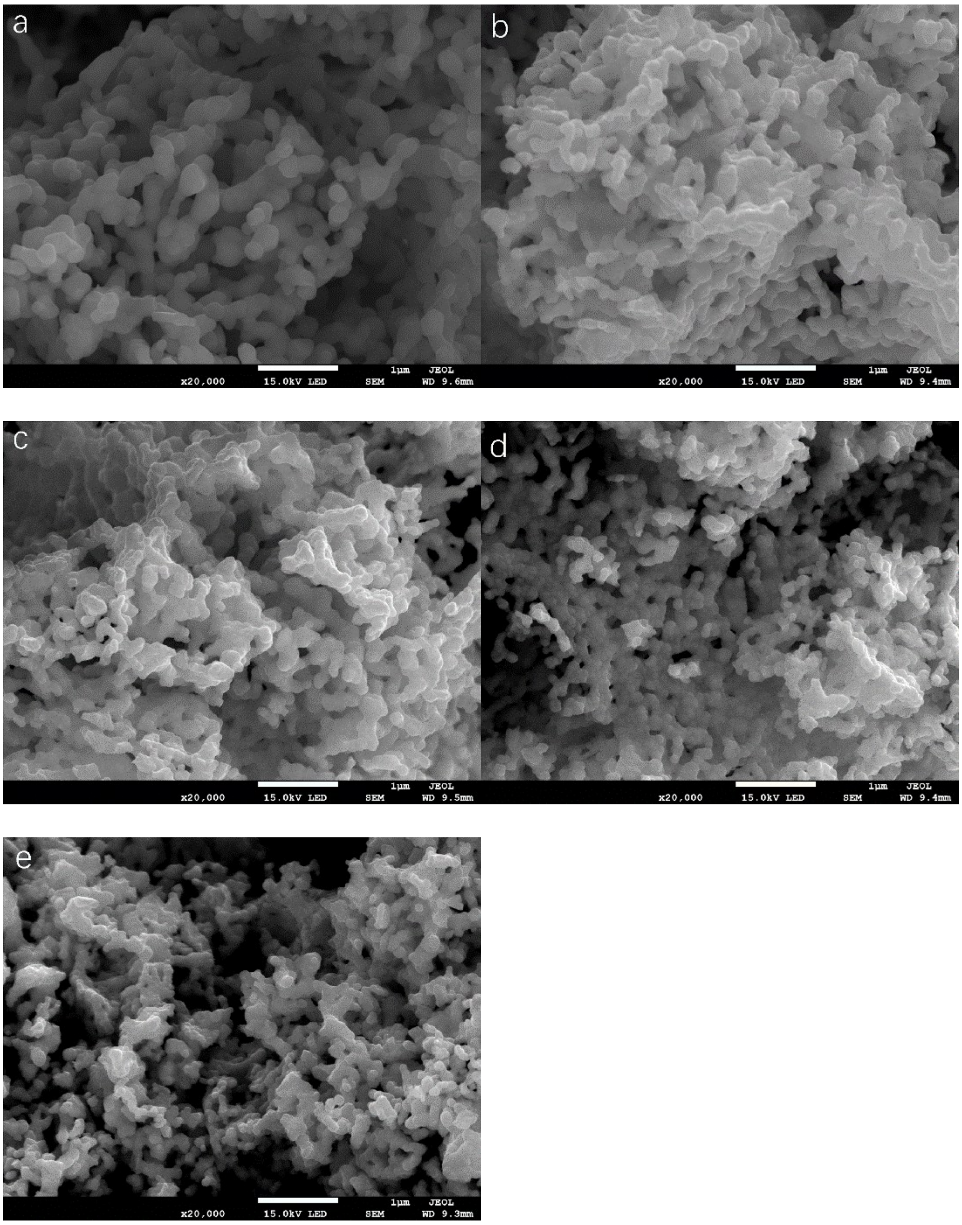

Figure S1. SEM images of a) $LaCoO_3$, b) $LaCo_{0.98}Pt_{0.02}O_{3-\delta}$, c) $LaCo_{0.96}Pt_{0.04}O_{3-\delta}$, d) $LaCo_{0.94}Pt_{0.06}O_{3-\delta}$ and e) $LaCo_{0.92}Pt_{0.08}O_{3-\delta}$ catalysts.

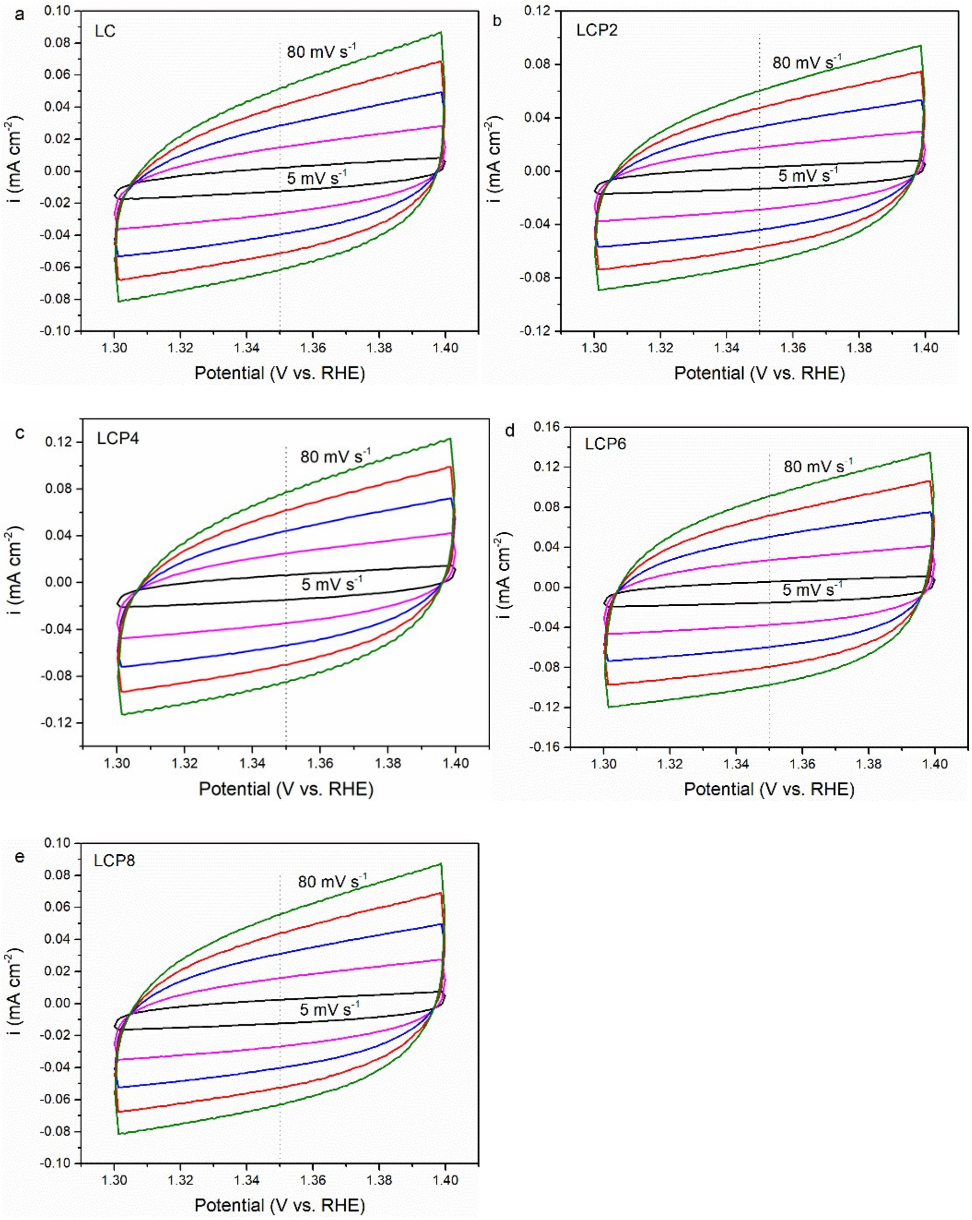

Figure S2. CV measurements in a non-faradic current region (0.3 to 0.4 V vs. Hg/HgCl2) at scan rates of 5, 20, 40, 60 and 80 mV s^{-1} of a) $LaCoO_3$, b) $LaCo_{0.98}Pt_{0.02}O_{3-\delta}$, c) $LaCo_{0.96}Pt_{0.04}O_{3-\delta}$, d) $LaCo_{0.94}Pt_{0.06}O_{3-\delta}$ and e) $LaCo_{0.92}Pt_{0.08}O_{3-\delta}$ catalysts in 0.1 M KOH solution.

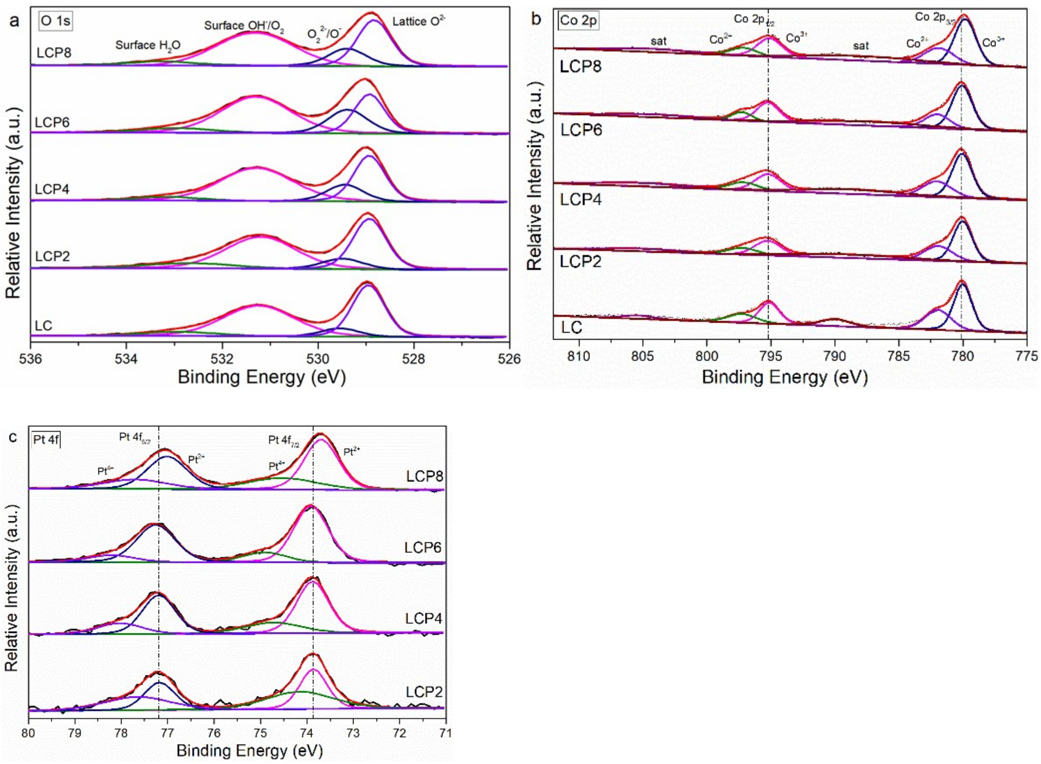

Figure S3. XPS spectra of a) O 1s, b) Co 2p and c) Pt 4f species on the surface of $LaCo_{1-x}Pt_xO_{3-\delta}$ (x = 0, 0.02, 0.04, 0.06, 0.08) powders.

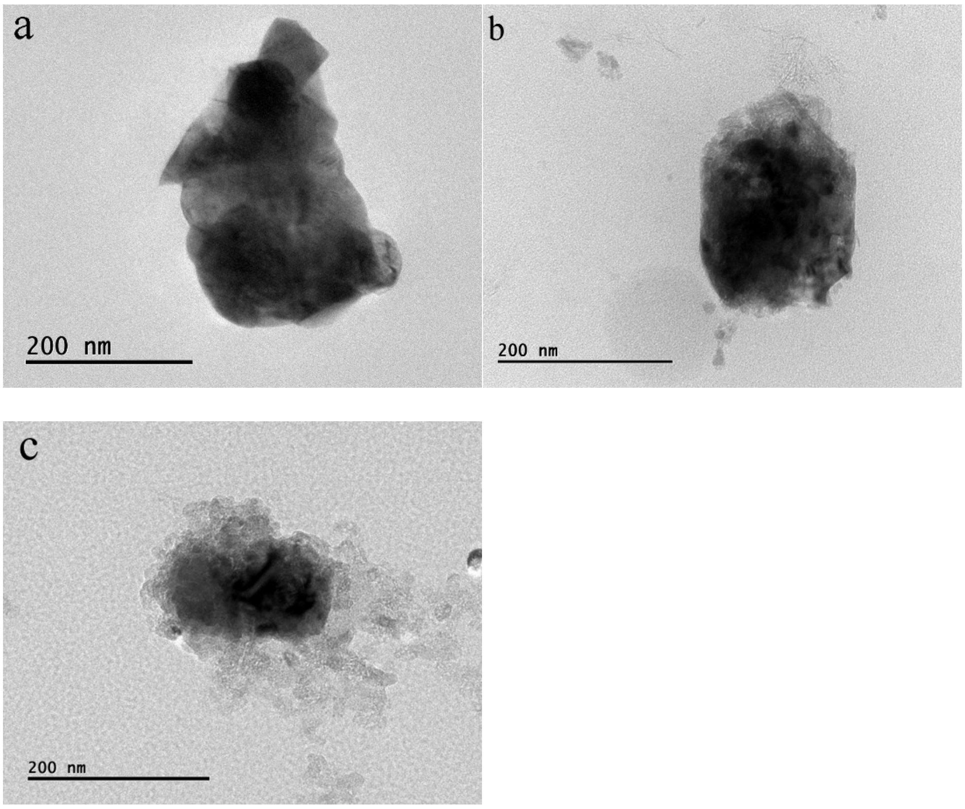

Figure S4. TEM images of $LaCo_{0.94}Pt_{0.06}O_{3-\delta}$ a) before the cyclic voltammetry for 500 cycles, b) after the cyclic voltammetry for 500 cycles in OER, C) after the cyclic voltammetry for 500 cycles in HER.

Table S1 A summary of perovskite electrocatalysts for OER.

Electrocatalysts	Electrolyte	η_{10} vs. RHE (mV)	Tafel slope (mV dec^{-1})	Reference
STFN/CNT-700	0.1 M KOH	~480	~98	Electrochim. Acta 2018, 286, 47-54
C-NSCFNb	1.0 M KOH	~420	~89	ChemElectroChem 2019, 6, 1520 –1524
SNCF-NR	0.1 M KOH	~390	~61	Adv. Energy Mater. 2017, 7, 1602122
L-0.5/rGO	1.0 M KOH	~285	~80	Adv. Energy Mater. 2017, 7, 1700666
PBSC@FeOOH-20	0.1 M KOH	~390	~53	ACS Appl. Mater. Interfaces 2018, 10, 38032−38041
3DOM-LFC82	1.0 M KOH	~410	~56	Adv. Mater. Interfaces 2019, 6, 1801317
NBM5.5	1.0 M KOH	~430	~75	ACS Catal. 2018, 8, 364−371
P-3G	0.1 M KOH	~320	~74	J. Mater. Chem. A, 2019, 7, 2048–2054
LCP6	0.1 M KOH	454	86	This work

Table S2 A summary of perovskite electrocatalysts for HER.

Electrocatalysts	Electrolyte	η_{10} vs. RHE (mV)	Tafel slope (mV dec^{-1})	Reference
SCFM0.05	0.1 M KOH	~323	~94	Electrochim. Acta 2019, 312, 128-136
STFN/CNT-700	0.1 M KOH	~340	~166	Electrochim. Acta 2018, 286, 47-54
C-NSCFNb	1.0 M KOH	~470	~133	ChemElectroChem 2019, 6, 1520 –1524
SNCF-NR	0.1 M KOH	~262	~134	Adv. Energy Mater. 2017, 7, 1602122
L-0.5/rGO	1.0 M KOH	~187(η_{150})	~46	Adv. Energy Mater. 2017, 7, 1700666
PBSC@FeOOH-20	0.1 M KOH	~280	~70	ACS Appl. Mater. Interfaces 2018, 10, 38032−38041
3DOM-LFC82	1.0 M KOH	~360	~111	Adv. Mater. Interfaces 2019, 6, 1801317
NBM5.5	1.0 M KOH	~290	~87	ACS Catal. 2018, 8, 364−371
P-3G	0.1 M KOH	~230	~124	J. Mater. Chem. A, 2019, 7, 2048–2054
LCP6	0.1 M KOH	294	148	This work
